# Biochemical and biomechanical properties of the pacemaking sinoatrial node extracellular matrix are distinct from contractile left ventricular matrix

**DOI:** 10.1371/journal.pone.0185125

**Published:** 2017-09-21

**Authors:** Jessica M. Gluck, Anthony W. Herren, Sergey Yechikov, Hillary K. J. Kao, Ambereen Khan, Brett S. Phinney, Nipavan Chiamvimonvat, James W. Chan, Deborah K. Lieu

**Affiliations:** 1 Division of Cardiovascular Medicine, Department of Internal Medicine, University of California Davis; Davis, CA, United States of America; 2 UC Davis Genome Center, University of California Davis; Davis, CA, United States of America; 3 Bridges to Stem Cell Research Program, California State University Sacramento; Sacramento, CA, United States of America; 4 Department of Veterans Affairs, Northern California Health Care System; Mather, CA, United States of America; 5 Center for Biophotonics, University of California Davis; Sacramento, CA, United States of America; 6 Department of Pathology and Laboratory Medicine, University of California Davis; Sacramento, CA, United States of America; University of Bergen, NORWAY

## Abstract

Extracellular matrix plays a role in differentiation and phenotype development of its resident cells. Although cardiac extracellular matrix from the contractile tissues has been studied and utilized in tissue engineering, extracellular matrix properties of the pacemaking sinoatrial node are largely unknown. In this study, the biomechanical properties and biochemical composition and distribution of extracellular matrix in the sinoatrial node were investigated relative to the left ventricle. Extracellular matrix of the sinoatrial node was found to be overall stiffer than that of the left ventricle and highly heterogeneous with interstitial regions composed of predominantly fibrillar collagens and rich in elastin. The extracellular matrix protein distribution suggests that resident pacemaking cardiomyocytes are enclosed in fibrillar collagens that can withstand greater tensile strength while the surrounding elastin-rich regions may undergo deformation to reduce the mechanical strain in these cells. Moreover, basement membrane-associated adhesion proteins that are ligands for integrins were of low abundance in the sinoatrial node, which may decrease force transduction in the pacemaking cardiomyocytes. In contrast to extracellular matrix of the left ventricle, extracellular matrix of the sinoatrial node may reduce mechanical strain and force transduction in pacemaking cardiomyocytes. These findings provide the criteria for a suitable matrix scaffold for engineering biopacemakers.

## Introduction

One in 600 cardiac patients who are 60 years or older has sinoatrial node (SAN) dysfunction, amounting to about half of the pacemaker recipients in the United States [1]. Implantable pacemakers are a common treatment for rhythm disorders but are costly, invasive, lack sympathetic response, and have limited battery life. Recent advances in cardiovascular tissue engineering hold promise for replacing dysfunctional SAN with a bioengineered pacemaker that may circumvent limitations associated with electronic devices. In particular, the extracellular matrix (ECM) is a critical component of a bioengineered cardiac tissue, by serving as a scaffold providing structural support and a physiological microenvironment that transmits signals to regulate the development and function of cardiac cells [2].

The native ECM should be the most conducive for differentiation and phenotype maintenance of its resident cells. To date, studies have only focused on the ECM of the contractile myocardium, specifically of the left ventricle (LV) [3–5], and there is a paucity of information on ECM of the SAN [6–9]. None has considered regional differences in the ECM between the pacemaking and contractile tissues. Determining the properties of the SAN ECM can improve our understanding of the pacemaking microenvironment that is the most appropriate for a bioengineered pacemaker, as well as the physiological and pathological development of the SAN.

The SAN serves as the native pacemaker of the heart and is located in the right atrium at the junction of the crista terminalis with the intercaval region bordered by the superior and inferior vena cava [6]. In this study, we isolated this region from porcine hearts and quantified the biochemical and biomechanical properties of its ECM in comparison to that of the LV, which is the most efficient contractile region and typically used in cardiac tissue engineering, using scanning electron microscopy (SEM), atomic force microscopy (AFM), mass spectrometry, and immunostaining. Decellularized porcine cardiac ECMs were chosen for their clinical potential to engineer xenografts since porcine-derived products, such as small intestinal submucosa and valves, have already been FDA-approved for clinical transplantation [10]. We found the ECM of the SAN to be both biochemically and biomechanically distinct from that of LV. Its unique properties may contribute to the development and the resulting phenotype of the resident pacemaking cardiomyocytes by protecting the resident cells from mechanical stress. The biomechanical and biochemical properties of ECM in the SAN that have been determined in this study provide a set of criteria for a suitable matrix scaffold for engineering biopacemakers.

## Materials and methods

### Isolation and decellularization of porcine SAN and LV

Fresh 6-month old market hog hearts were obtained from the UC Davis Meat Laboratory. The SAN was identified and manually dissected under a microscope (panel A in S1 Fig). The SAN region was verified by hematoxylin/eosin staining (panel A in S1 Fig) as well as immunostaining for hyperpolarization-activated cyclic nucleotide-modulated (HCN)4 channels to identify the pacemaking cardiomyocytes. For all experiments, the LV served as the control. To decellularize the SAN and LV for analysis by SEM, AFM, and mass spectrometry, the pieces were incubated in 10mM Tris with 0.1% EDTA for 1 hour, then exposed to a hypotonic solution (0.1X PBS) for 3 hours, followed by 3 hours in a hypertonic solution (10x PBS). Next, the tissue pieces were incubated in 0.5% SDS for 6 hours, with the solution being refreshed every 2 hours. Finally, the pieces were then treated with DNase I for 10 min at 37°C. A rocker plate was used to agitate the tissue pieces for the entire procedure. The decellularized matrices were stored at 4°C in the PBS with 5% penicillin and streptomycin. Visually, the decellularized matrices appeared transparent for the contractile regions in the LV and the right atrium but whitish and opaque for the SAN area (panel A in S1 Fig). Thorough decellularization was verified by the absence of nuclear stain with Hoechest 33342 (panel B in S1 Fig).

### SEM assessment of ECM ultrastructure

Decellularized SAN and LV (n = 4 each) were fixed in Karnovsky’s solution and processed for SEM by the Electron Microscopy Laboratory in the Department of Pathology and Laboratory Medicine in the UC Davis Health System. Samples were mounted onto stubs and sputter coated with gold/palladium (Au/Pd to a thickness of ~10μm) using Pelco Auto Sputter Coater SC-7 (Ted Pella) before scanning with a Philips XL30 TMP (FEI Company) using image analysis iTEM software. Images of the LV matrix were analyzed for pore size of pores formed at the interstices of the fibers using ImageJ software. For each sample, at least 5 scanning electron micrographs at 1000x magnification were used for image analysis and pore size measurement.

### AFM assessment of ECM mechanical stiffness

Stiffness of the porcine SAN and LV matrix scaffolds were measured using a nano-indentation method as previously described [11, 12]. Decellularized SAN and LV were processed as described above and then mounted to glass bottom dishes (50/40mm, Wilco Wells) using waterproof double-sided tape (Nitto Denko America). AFM probes with silicon nitride cantilever of spring constant 0.06 N/m and 5 μm borosilicate spherical glass tip (Novascan) were used. Force maps were collected in an 8x8 array spanning a 5x5 μm^2^ area using a MFP-3D AFM (Asylum Research) at the UC Davis Keck Spectral Imaging Facility. Data was analyzed using Asylum Research software using the elastic Hertz model. An assumed Poisson’s ratio of 0.45 was used for analysis [11, 12]. All results are presented as mean ± standard error of the mean, with n = 435 for the SAN from three hearts and n = 637 for the LV from six hearts. Statistical significance was determined using a Students’ t-test analysis. P-values < 0.05 were considered as statistically significant.

### Preparation and proteomic analysis of cardiac ECMs

An expanded methods section of the proteomic analysis is presented in the supplement (S1 File). Isolated SAN and LV were pooled from four porcine hearts. The tissues were first decellularized, then ECM proteins were extracted based on a modified version of a previously described protocol [13]. Briefly, approximately 150 mg of pooled ECMs were solubilized in 4M guanidine hydrochloride (GuHCL), pH 6.8, for 72 hours at room temperature on vortex to produce two protein fractions—GuHCl-soluble and GuHCl-insoluble—for each SAN and LV. The GuHCL-soluble fraction was further deglycosylated and protein precipitated. Resultant proteins for all samples were resuspended in urea, reduced in dithiothreitol (DTT), alkylated in iodoacetamide, and proteolytically digested in solution under denaturing conditions with sequencing grade Lys-C/Trypsin (GuHCL-soluble and GuHCL-insoluble) or Elastase (GuHCL-insoluble) (Promega). Digested peptides from each fraction were acidified with trifluoroacetic acid (TFA), concentrated and desalted with a C18 resin and analyzed by liquid chromatography-mass spectrometry/mass spectrometry (LC-MS/MS) on a Thermo Scientific Exactive Plus Orbitrap Mass Spectrometer in conjunction with a Thermo EASY-nLC 1200 UHPLC and custom rigged Proxeon nanospray source.

Tandem mass spectra were extracted and charge state deconvoluted and searched using the Andromeda (MaxQuant framework, Max-Planck Institute) or X! Tandem (The GPM, thegpm.org) peptide spectrum matching algorithms against a *Sus scrofa* protein database (uniprot.org). Data were validated with false discovery rates and protein/peptide identification probabilities generated using a target-decoy database approach, as well as by manual inspection (Scaffold, Proteome Software). The relative protein contribution within each tryptic sample was quantified by label-free methods using chromatographic ion intensities extracted with MaxQuant and normalized by intensity based absolute quantification (IBAQ), as previously described [14]. Relative abundance is presented for identified ECM proteins within SAN or LV as a percentage of the total identified ECM. Mass spectrometry data has been deposited into the MassIVE repository (https://massive.ucsd.edu/ProteoSAFe/static/massive.jsp) under submission ID MSV000081000.

### Immunofluorescent staining of the SAN and LV tissues for ECM proteins

Fresh SAN and LV tissues in OCT compound were cryosectioned into 10 μm slices and fixed with 4% paraformaldehyde for immunofluorescent staining of ECM proteins—collagen I (ab34710, Abcam), collagen III (ab7778, Abcam), collagen IV (ab6586, Abcam), elastin (ab21610, Abcam), fibronectin (ab2413, Abcam), Laminin (ab30320, Abcam)—and pacemaking cardiomyocyte marker HCN4 (75–150, NeuroMab) with Alexa Fluor 647 goat anti-mouse IgG1 antibody, Alexa Fluor 488 goat anti-rabbit IgG antibody and nuclear counterstain Hoechst33342 (Molecular Probes). Slices mounted with ProLong Gold antifade mounting medium (Molecular Probes) were imaged using a Nikon A1 confocal microscope.

## Results

### Ultrastructure of the SAN ECM is distinct from that of the LV

**Fig 1:**
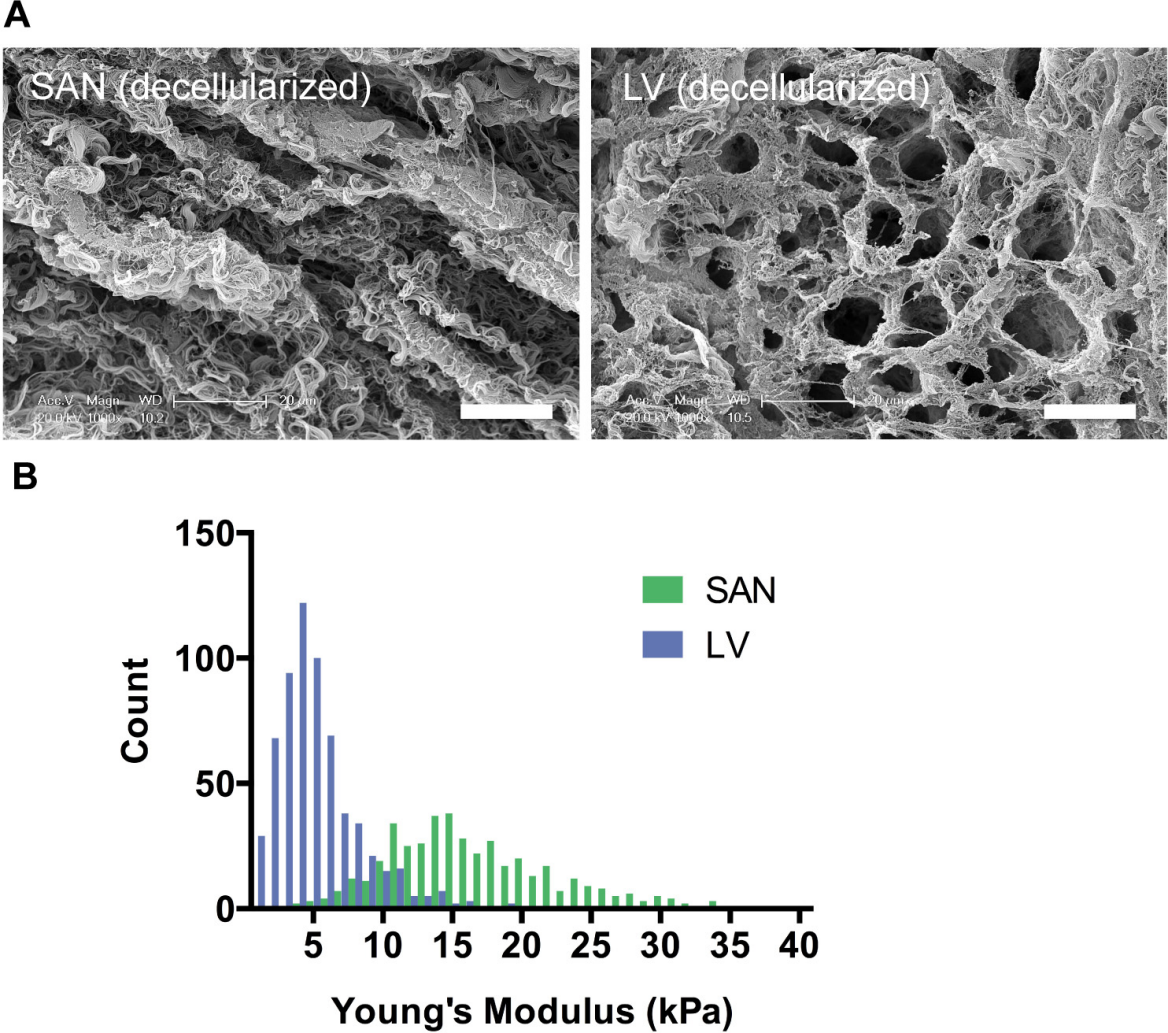
Ultrastructure and stiffness of decellularized porcine SAN relative to LV. **A)** SEM images of decellularized pacemaking SAN showed a rope-like fibrous ECM compared to a mesh-like ECM in the contractile LV. (Scale bars: 20 μm) **B)** Young’s modulus measured by AFM was higher for decellularized SAN than LV (p<0.0001) indicating a stiffer matrix in the SAN than the LV.

To assess the ultrastructure of ECM in the SAN relative to LV, SEM images of decellularized ECMs were acquired from four porcine hearts. Interestingly, the decellularized SAN and LV matrices exhibited very different ultrastructure (Fig 1A). The SAN matrix scaffold exhibited a rope-like ultrastructure with entwined fibrous strands. In contrast, the decellularized LV appeared to be a porous mesh with fewer visible fibrous strands and distinctive pores that were previously occupied by cardiomyocytes and other cardiac cells. The median pore size for LV was about ~52 μm^2^ with an interquartile range of 32–86 μm^2^. The pore size was not obviously quantifiable for the SAN since only grooves that appeared to be spaces devoid of cells in the matrices were present. The matrix also appeared to be denser, less organized, and more fibrous for the SAN than the LV.

### The SAN ECM is stiffer than the LV ECM

To determine stiffness of the matrix from the SAN relative to the LV, several regions of the decellularized matrix from the SAN and the LV were assessed for the Young’s Modulus using an AFM (Fig 1B). Young’s modulus is a stiffness parameter that quantifies the ratio of imposed stress to the resulting strain in a material where an elastic material would have a lower Young’s modulus than that of a stiffer material. In the absence of the cardiac cells, the LV matrix scaffold has an average Young’s modulus of 5.36±0.14 kPa. Comparatively, the SAN ECM was stiffer with a significantly higher average Young’s modulus of 16.69±0.32 kPa.

### The SAN ECM differs in biochemical composition from the LV ECM

**Fig 2:**
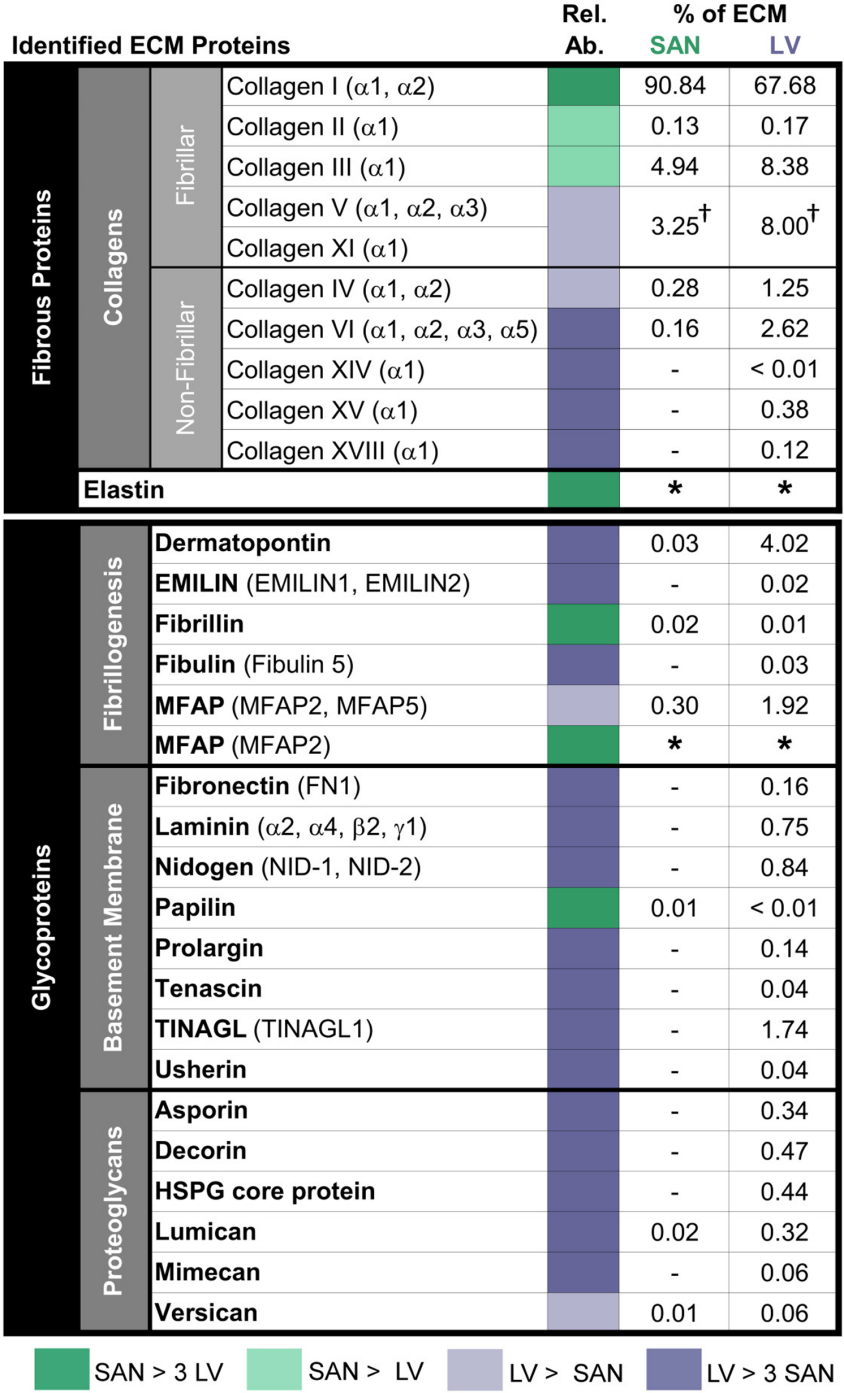
Mass spectrometry-identified ECM proteins from decellularized porcine SAN and LV. Each ECM protein is presented as a percentage of the total ECM protein detected. Comparisons of relative protein abundance (Rel. Ab.) between SAN and LV are encoded in color. SAN is predominantly comprised of hydroxylated collagen I whereas LV is comprised of a greater and more abundant distribution of fibrillar and non-fibrillar collagen types as well as glycoproteins. Elastin and MFAP were detected in SAN but not LV. Green indicates more abundance of proteins in the SAN than the LV, while purple indicates more abundance of proteins in the LV than the SAN, where a darker coloring shade means the abundance is more than 3 times higher. All protein values are calculated from tryptic digests of GuHCl soluble fractions. † denotes merging of proteins due to high homology leading to indistinguishable **α**-subunits. * denotes proteins detected separately from GuHCl-insoluble ECM fractions digested by elastase rather than trypsin, therefore, the percentage to total ECM proteins could not be computed. No value means none detected.

**Fig 3:**
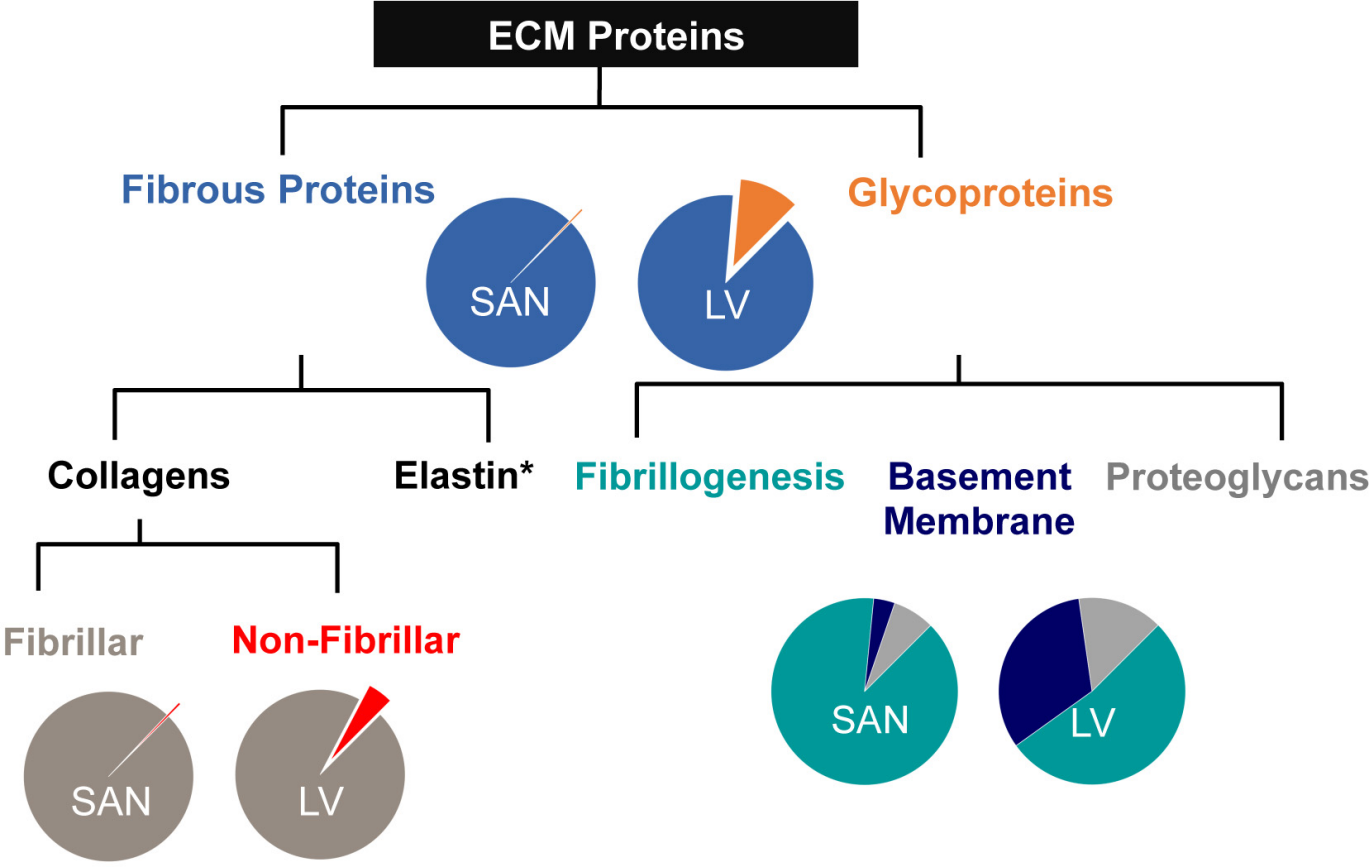
ECM protein composition of porcine SAN relative to LV ECM. ECM proteins of decellularized SAN and LV assessed by mass spectrometry were classified by categories. SAN matrix in contrast to its contractile counterpart was composed mostly of fibrous proteins that were predominantly fibrillar collagens. The small fraction of glycoproteins present was mostly proteins associated with fibrillogenesis of collagens. Basement membrane-associated proteins contributed to a very minute fraction in the SAN ECM.

To determine the biochemical composition of ECM in the SAN relative to LV, decellularized SAN and LV were assessed by a label-free mass spectrometry-based approach to identify and characterize the relative abundance of GuHCl-soluble ECM proteins within each matrix. Out of the detected ECM proteins, 14 and 29 different ECM proteins were detected in the SAN and LV, respectively, with 13 overlapping proteins (Fig 2). We classified the detected ECM proteins into fibrous proteins and glycoproteins according to the literature [2]. Fibrous proteins accounted for majority of the ECM proteins in the SAN similar to the LV matrices (Fig 3). Glycoproteins did encompass a much smaller fraction of the total ECM proteins in the SAN relative to the LV. As expected, collagens were by far the most abundant family of ECM proteins found in both the SAN and LV, representing nearly 100% and 89% of the total ECM proteins, respectively, and were heavily hydroxylated on proline and lysine residues. The fibrillar collagens accounted for majority of the fibrous proteins in both the SAN and LV, but LV had a greater and more abundant distribution of collagen types within more non-fibrillar collagens and fibrillar collagens other than collagen I (Figs 2 and 3). Collagen I was the most abundant collagen type by far representing ~91% of the total ECM protein for the SAN and ~76% for the LV. Subunits **α**1 and **α**2 for collagen I were both detected by mass spectrometry with the amount of **α**1 more than doubled that of **α**2 as expected for an **α**1**α**1**α**2 composition. Fibrillar collagens III, the next most abundant collagen, was also nearly 42% more abundant in the SAN relative to the LV, whereas the less abundant non-fibrillar collagens IV and VI were found to be about 2- and 7-folds higher in the LV. Collagens XIV, XV, and XVIII were exclusively identified in the LV.

In addition to collagens, the fibrous protein elastin was also detected in the SAN with a primary protein sequence coverage of 26% in contrast with no detection in the LV. Because elastin is known to exist in insoluble protein fractions due to its high hydrophobicity and is resistant to proteolytic digestion with trypsin (owing to its sequence), we digested GuHCl-insoluble fractions with elastase, for which elastin is a natural substrate. Its contribution to the total ECM proteins was not included in Fig 2 since it was detected separately from the other ECM proteins identified in tryptic GuHCl-soluble fractions. Proteotypic elastin peptides and a representative elastin peptide spectrum are presented in S6 Fig and S1 Table.

In addition to differences in fibrous proteins, various glycoproteins known to be important in the formation and composition of the basement membrane were less abundant in the SAN compared to the LV. Glycoproteins were divided into subcategories—fibrillogenesis-associated, basement membrane-associated, and proteoglycans—based on the classification in the literatures [2, 15, 16]. The fibrillogenesis-associated proteins were the most abundant glycoproteins by far in the SAN at 89% but account for only 53% in the LV ECM that comprised of a sizable fraction of basement membrane-associated proteins at 33% followed by proteoglycans at 15% (Figs 2 and 3). Most of the glycoproteins detected in the SAN are involved in fibrillogenesis of either elastin (fibrillin and microfibril-associated proteins (MFAPs)) or collagens (dermatopontin and lumican), with MFAPs being the most abundant glycoproteins. It is important to note that although MFAPs comprised a significant portion of the LV, more MFAP2 was found in the GuHCl-insoluble fraction of the SAN ECM (presumably associated with elastin). Next to collagens, the fibrillogenesis-associated glycoprotein dermatopontin was the most abundant ECM protein overall and was found over 100-fold higher in LV.

Basement membrane-associated glycoproteins and proteoglycans important for adhesion and signaling were more abundant in the LV matrix relative to the SAN (Fig 2). Those basement membrane-associated glycoproteins involved in cell adhesion, such as fibronectin, laminin and nidogens, or the formation and composition of the basement membrane, such as usherin and prolargin, were all exclusively detected in the LV in contrast to the SAN. Matricellular proteins—tenascin and tubulointerstitial nephritis antigen-like (TINAGL)—were also found to be below detectable level in the SAN matrix. In addition, proteoglycans important for ECM lattice structure and spacing were more abundant or exclusively detected in the LV, which include small leucine rich proteoglycans (SLRPs), such as asporin, decorin, lumican, and mimecan, as well as versican, a known hyalectan, and heparin sulfate proteoglycan (HSPG) core protein.

### Biochemical distribution of ECM proteins differs between the pacemaking and contractile tissue

**Fig 4:**
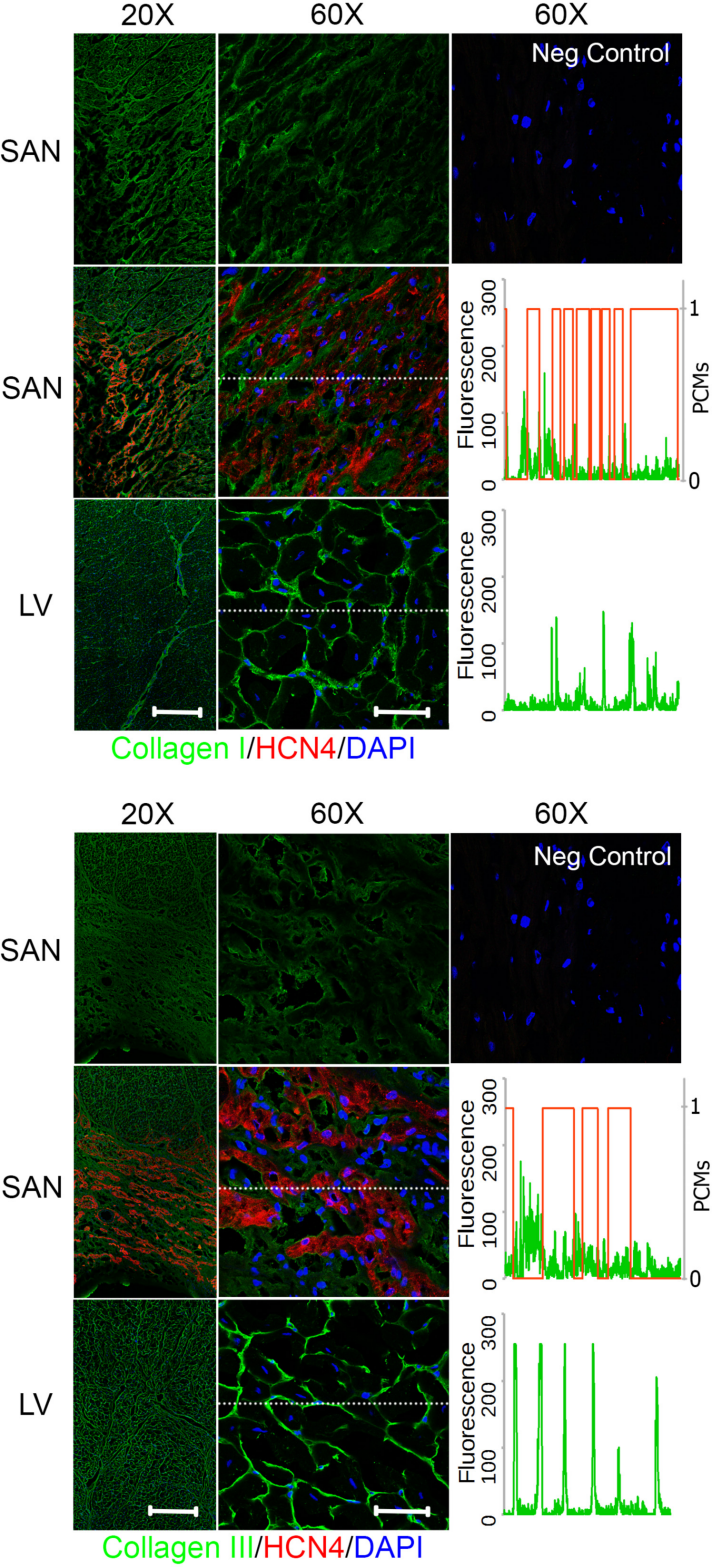
Fibrillar collagen distribution in the SAN relative to LV. Right atrium containing SAN and LV were immunostained for collagen I (*top*) or collagen III (*bottom*) and hyperpolarization-activated cyclic nucleotide-activated (HCN)4 channels for identification of the SAN region with pacemaking cardiomyocytes. The images in the top row for each protein show the SAN staining in a single channel for the specified ECM protein only. Fluorescence intensity for region marked by a dotted white line in the 60X SAN and LV images were plotted against HCN4 fluorescence in binary scale such that 1 indicates presence of pacemaking cardiomyocytes (PCMs) and 0 for no pacemaking cardiomyocytes. Both collagens showed higher fluorescence in the perimysium between clusters of pacemaking cardiomyocytes in the SAN. Scale bars: 400 μm (20X) and 50 μm (60X).

**Fig 5:**
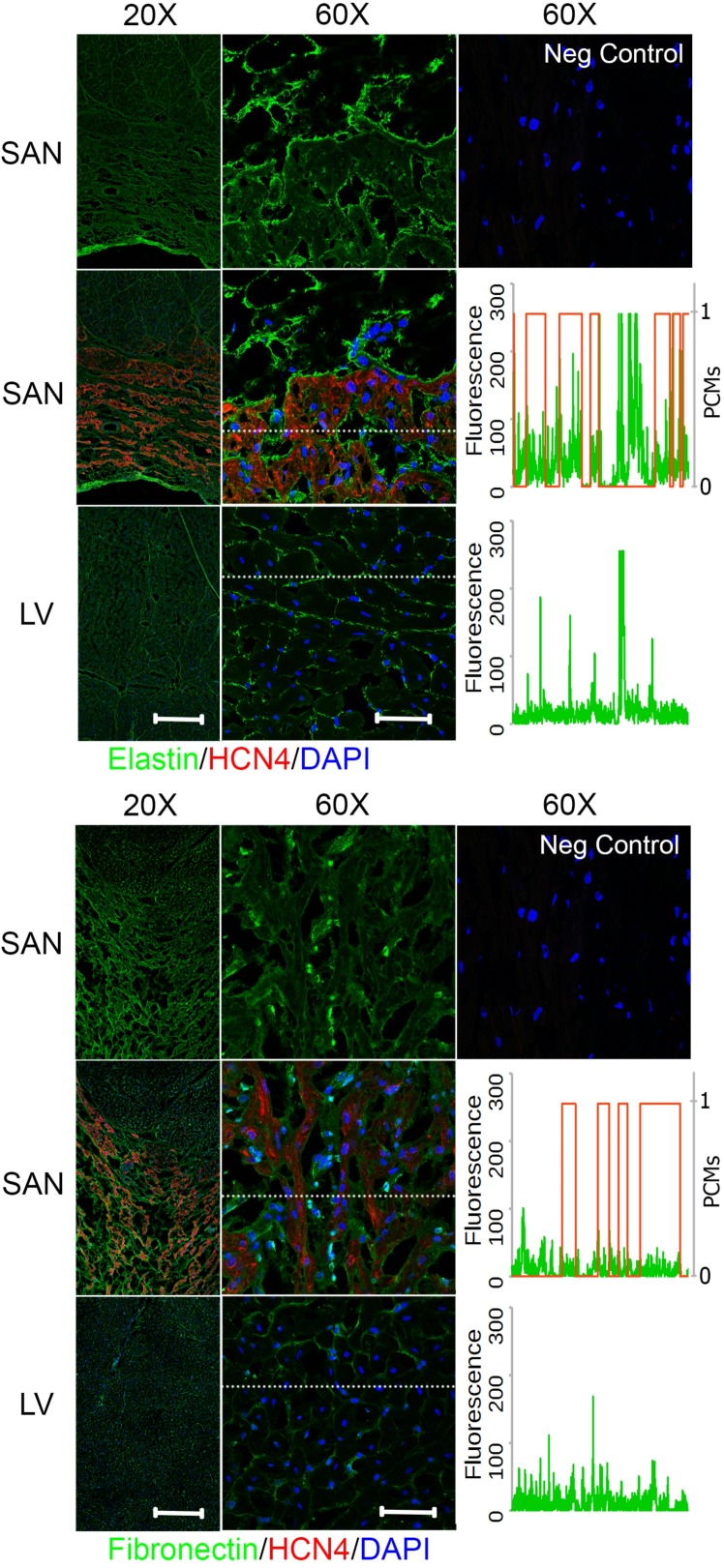
Elastin and fibronectin distribution in the SAN relative to LV. Right atrium containing SAN and LV were immunostained for elastin (*top*) or fibronectin (*bottom*) and hyperpolarization-activated cyclic nucleotide-activated (HCN)4 channels for identification of SAN region with pacemaking cardiomyocytes. The images in the top row for each protein show the SAN staining in a single channel for the specified ECM protein only. Fluorescence intensity for region marked by a dotted white line in the 60X SAN and LV images were plotted against HCN4 fluorescence in binary scale to indicate 1 or 0 for pacemaking cardiomyocytes (PCMs) or no pacemaking cells, respectively. Elastin was predominantly in the perimysium between clusters of pacemaking cardiomyocytes in the SAN. Fibronectin was in similarly distributed between the perimysium and endomysium surrounding the pacemaking cardiomyocytes. Scale bars: 400 μm (20X) and 50 μm (60X).

**Fig 6:**
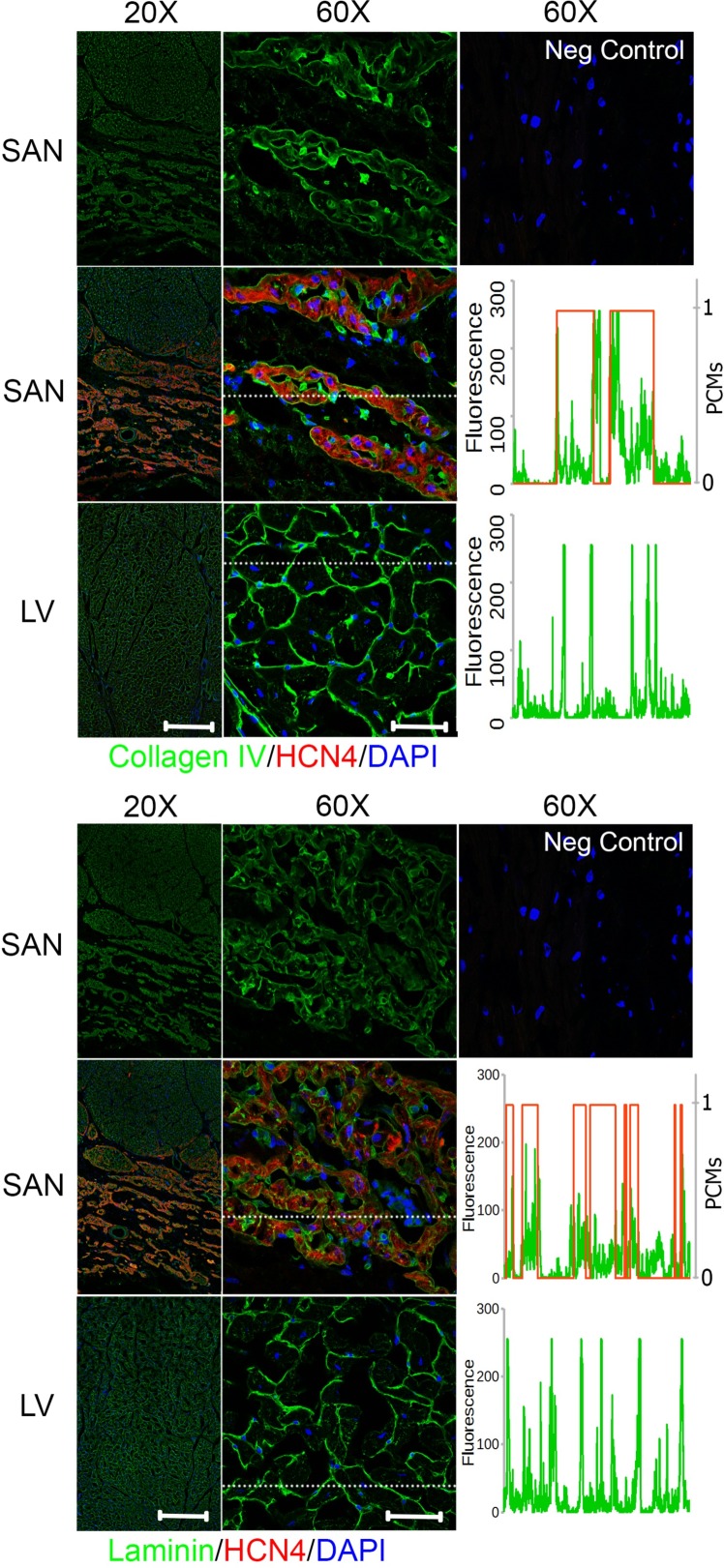
Basement membrane-associated adhesion protein distribution in the SAN relative to LV. Right atrium containing SAN and LV were immunostained for collagen IV (*top*) or laminin (*bottom*) and hyperpolarization-activated cyclic nucleotide-activated (HCN)4 channels for identification of SAN region with pacemaking cardiomyocytes. The images in the top row for each protein show the SAN staining in a single channel for the specified ECM protein only. Fluorescence intensity for region marked by a dotted white line in the 60X SAN and LV images were plotted against HCN4 fluorescence in binary scale to indicate 1 for pacemaking cardiomyocytes (PCMs) and 0 for no pacemaking cells. Both adhesion proteins were predominantly present in the endomysium surrounding each pacemaking cardiomyocytes in the SAN. Scale bars: 400 μm (20X) and 50 μm (60X).

To investigate the biochemical distribution of the ECM proteins in the SAN relative to LV, cryosections exhibiting cross-sectional and longitudinal orientations of cardiomyocytes from regions of the right atrium that included the SAN and the LV (section orientation in S2 Fig) were immunostained for ECM proteins—collagen I, III, IV, elastin, fibronectin, or laminin—in addition with HCN4 for identifying the SAN region. In the cross-sectional right atrial images, the SAN that was positive for HCN4 below a layer of atrial myocardium could be clearly identified (Figs 4–6). To better understand the ECM protein distribution relative to the pacemaking cardiomyocytes in the SAN, a representative spatial fluorescence intensity of each ECM protein (green) extracted from a high magnification cross-sectional image was plotted against a binary scale for HCN4 (red) indicating the presence of pacemaking cardiomyocytes (1 for presence of pacemaking cardiomyocytes and 0 for no pacemaking cell) (Figs 4–6). An ECM protein distribution for the LV was similarly plotted for comparison. The contractile cardiomyocytes in the LV were aligned with each other and can be clearly seen in the cross-sectional (Figs 4–6) and longitudinal images (S3 Fig), but a major axis of alignment was less distinct in the SAN than the LV.

### Fibrillar collagens

In both the SAN and LV, fibrillar collagens I and III were present in the endomysium that surrounds individual cardiomyocytes as well as the perimysium surrounding bundles of cardiomyocytes (Fig 4). Comparing within the SAN or LV, the fluorescence intensity of collagen I appeared to be higher in the perimysium than the endomysium, while the fluorescence intensity of collagen III was mostly comparable between the perimysial and endomysial space. The fluorescence intensity of collagen I in the perimysial region was comparable between the SAN and LV while perimysial collagen III seems to be generally lower for the SAN than the LV. This is better illustrated by the fluorescence intensity plots across a region of interest showing an inverse relationship between collagen I and the presence of pacemaking cardiomyocytes in the SAN as indicated by HCN4 staining, while this pattern was less evident for collagen III. Nevertheless, both fibrillar collagens were distributed over the perimysial space that was absent of cardiomyocytes. Interestingly, perimysium surrounding cardiomyocyte aggregates in the SAN encompassed a much larger space than that of the LV, such that the pacemaking cell clusters appeared as islands in the expansive perimysium composed of fibrillar collagens (Fig 4). Notably, the organization of the SAN matrix resembles the epicardial connective tissue more than the contractile muscle regions (S4 Fig).

### Elastin

An abundance of elastin could be observed in the perimysial space surrounding the cardiomyocyte aggregates in the SAN as well as the space spanning the cell aggregates but much lower levels were observed in the endomysium (Fig 5). This preferential localization in the perimysium in both the SAN and LV was similar to the distribution pattern of fibrillar collagens. Overall, the florescence intensity of elastin in the endomysium of the SAN was greater than that in the LV but comparable in the perimysium. However, the total abundance of elastin in the perimysium was greater in the SAN than LV due to a greater perimysial space in the SAN than LV.

### Basement membrane-associated ECM proteins

Basement membrane is the endomysial ECM that immediately surrounds the cardiac cells. ECM proteins associated with this matrix scaffold include non-fibrillar collagen IV and glycoproteins—fibronectin and laminin. Compared to the distribution of fibrillar collagens and elastin, the fibronectin staining was mostly restricted in the endomysium of the SAN and LV, such that there was staining in the pacemaking cell clusters but a diminished fluorescence of fibronectin at the border surrounding islands of pacemaking cardiomyocytes (Fig 5). The fluorescence intensity of fibronectin staining was generally comparable between the SAN and LV as demonstrated in the fluorescence plots.

Collagen IV and laminin shared a similar staining pattern in both the SAN and LV (Fig 6). The ECM proteins were present exclusively in the endomysium. There was a minimal presence of collagen IV and laminin observed between the aggregates of pacemaking cardiomyocytes and surrounding the non-cardiomyocytes. While the endomysial level of collagen IV was comparable between the SAN and LV, the abundance of laminin was lower in the SAN relative to the LV as shown in the fluorescence plot.

Overall, the endomysium appeared to encompass less space while the perimysium was more expansive in the SAN compared with the LV. The ECM protein distribution of decellularized SAN and LV that were used for SEM, AFM, and mass spectrometry also showed similar staining patterns to the native tissues (S5 Fig).

## Discussion

Although human SAN sections have been histologically evaluated and optically mapped providing an understanding of the pacemaking cardiomyocyte organization and electrophysiology [17–19], studies characterizing the composition and mechanical properties of the pacemaking microenvironment are lacking. The understanding of the role of the ECM has expanded recently from mere structural and mechanical support to providing force transmission and presenting signaling molecules to the resident cardiac cells. Information on the ECM from the pacemaking region not only provides insights on the microenvironment that facilitate the development and maintenance of pacemaking cardiomyocytes but also establishes a critical foundation for engineering biopacemakers with a set of criteria for the ideal matrix scaffold to support the pacemaking cardiomyocytes. In this study, we demonstrated that both the biochemical and biomechanical properties of the pacemaking SAN matrix are distinct from that of the contractile LV matrix by examining the ultrastructure with SEM, the elastic properties using AFM, the biochemical composition of the ECM proteins using mass spectrometry, and the biochemical distribution of several prominent ECM proteins through immunostaining.

### Decellularized LV ECM as a contractile control for pacemaking SAN ECM

Although ECM protein composition from the SAN has not been assessed by mass spectrometry, ECM proteins from the LV of porcine origin have previously been reported [20, 21]. Comparing to the reported list of ECM proteins detected by mass spectrometry from the porcine LV by Johnson *et al*. [20], we detected all the proteins reported—collagen I-VI, fibulin, fibrillin, fibronectin, laminin, and lumican—in the porcine LV by mass spectrometry with the exception of elastin. Notably, Barallobre-Barreiro *et al*. also did not detect elastin in their porcine LV samples [21]. Given that elastin is a highly hydrophobic and insoluble protein, its absence in the GuHCl-soluble fraction as we and Barallobre-Barreiro *et al*. had observed is not surprising. However, we did identify elastin in elastase-treated GuHCl-insoluble SAN but not the LV ECM in our study. Additionally, we have detected glycoproteins that were not in the reported list of proteins by Johnson *et al*. but were detected by Barallobre-Barreiro *et al*. Those included fibrillogenesis-associated proteins (asporin, decorin, dermatopontin, lumican, mimecan, versican), elastic or microfibril assembly-associated proteins (Elastin microfibril interface (EMILIN), MFAP), basement membrane associated proteins (HSPG core protein, nidogen, papillin, prolargin, ursherin), and matricellular protein (tenascin).

As previously described, immunostaining of the ECM surrounding contractile cardiomyocytes in the LV showed a tree-like hierarchy with a dense interstitial matrix surrounding bundles of cardiomyocytes called the perimysium and a basement membrane matrix surrounding the cells known as the endomysium [22, 23]. Consistently with a previous study, the perimysium of the LV was consisted of predominantly fibrillar collagens and elastin while the endomysium of the LV was present as a matrix of basement membrane in immediate contact with the cardiomyocytes [22]. This ECM included glycoproteins, such as fibronectin, laminin, non-fibrillar collagen IV, and proteoglycans [22]. In general, the ECM proteins detected in our decellularized porcine LV matrix by mass spectrometry and immunostaining are largely similar to previous reports. It is important to note that the decellularization treatment does lead to some inevitable loss of ECM proteins. Therefore, discrepancies that exist between our detected ECM proteins and those previously reported may be attributed to the differences in sample handling such as the decellularization protocol. In addition, the mechanical stiffness may be altered due to the loss of ECM proteins. A study using a largely similar decellularization protocol has demonstrated negligible loss of collagen but substantial loss of GAG and elastin at 19% and 27% of the native content, respectively [4]. Considering that majority of the ECM proteins are collagens (~100% for SAN and 89% for LV) and collagens are the major contributors of ECM stiffness, the effects of decellularization on ECM stiffness may be negligible. Moreover, immunostaining of major ECM proteins in the native and decellularized tissues demonstrated that the relative abundance of ECM proteins in the native cardiac tissues (SAN vs. LV) was mostly retained in the decellularized matrix scaffolds. Collectively, our data suggest that, the porcine LV matrix is a reasonable contractile control for a comparison against the pacemaking matrix.

It is important to note that although pigs and humans have similar cardiac physiology with comparable heart rate and cardiac output [24, 25], minor differences between the human and porcine LV ECMs have been reported [20]. Specifically, collagens II, V, and VI were identified only in the porcine and not the human LV ECM, but the relative abundance of ECM proteins between the species was not determined. However, the mechanical properties and ultrastructure of the LV ECM for the two species are similar, as demonstrated by comparable complex viscosity over several magnitudes of shear rate and SEM images, respectively. Because of the similar physiology and LV ECM properties between porcine and human, these findings in porcine SAN ECM are likely translatable to that of human and reveal the potential utility for porcine ECM as a source of xenotransplant material.

### Fibrillar collagens confer stiffness to the SAN matrix scaffold

Although collagen I (the most abundant collagen identified) was preferentially localized in the perimysium compared to the endomysium with comparable peak fluorescent staining intensity in these regions between the SAN and the LV, the perimysial space observed was more expansive in the SAN than the LV. This indicates a greater total abundance of the fibrillar collagen I in the SAN than the LV, which is in agreement with the assessment by mass spectrometry.

Collagen I is the stiffest fibrillar collagen whereas collagen III has been reported to confer more flexibility to the collagen fibrils [26]. Additionally, collagens III, V, and XI can limit fibril diameter through steric effects or increased rate of fibril nucleation [26, 27]. As such, a SAN matrix with a 2 to 5-fold higher ratio of Collagen I to III, V, and XI than that of the LV suggests that it may withstand greater tensile forces. Moreover, the SAN has fewer SLRPs (e.g. asporin, decorin, lumican, mimecan) that have inhibitory effects on collagen fibril assembly and fibril diameter [15, 26]. A greater abundance of fibrillar collagens in the SAN relative to LV is in agreement with ultrastructure observed with SEM showing dense rope-like fibrils. High fibril density was also reflected in a higher measured Young’s modulus by AFM in the decellularized SAN matrix than the LV. Hence, clusters of pacemaking cardiomyocytes were surrounded by dense fibrillar collagens that may better structurally shield and support them from mechanical stress.

### Elastin is interspersed among clusters of pacemaking cardiomyocytes in the SAN

Elastin, the only fibrous protein not in the collagen family and the main component of elastic fibers, was detected solely in the elastase-digested GuHCl-insoluble fraction of the SAN ECM. This is due to its hydrophobic amino acid-rich composition rendering it resistant to trypsinization and highly insoluble under most extraction conditions [28]. No detectable elastin in the LV ECM by mass spectrometry does not suggest that it was completely absent, but rather that its level in the decellularized LV was below detection threshold. It does suggest, however, that the abundance of elastin in the SAN ECM was likely much higher than that of LV. In addition, its presence in the GuHCl-insoluble fraction was accompanied by MFAP, which is responsible for elastic fiber assembly. Together with the identification of more fibrillin in the SAN, which forms the microfibril sheath surrounding the elastin core [28], our observations suggest that there are more elastic fibers present in the SAN than the LV. Elastin immunostainings of the SAN and LV support this finding as a fibrous pattern of elastin was observed in the perimysium for both cardiac matrices but the spatial coverage was much greater in the SAN due to a more expansive perimysium. Contrary to a mere thin layer around bundles of cardiomyocytes in the LV, elastin filled large regions of the perimysial areas between the islands of pacemaking cardiomyocytes in the SAN.

Elastic fibers, enabling a matrix to stretch and recoil upon mechanical stress, would naturally lower the stiffness of a matrix, but regions high in elastin is not inconsistent with a higher measured Young’s modulus (*E*) in the SAN. Components of ECM proteins contribute to the overall mechanical properties of a matrix scaffold forming a composite material as described by the equation *E* = *∑ V_i_E_i_*, where *V*_*i*_ is the fractional volume and *E*_*i*_ is the Young’s modulus for a material component *i* in the composite material [29]. Hence, regions of high and low stiffness contribute in a weighted fashion to a global stiffness. For the same imposed mechanical stress, regions of differing Young’s modulus respond with varying strain. Elastins that are two orders of magnitude more elastic than the fibrillar collagens [30] may reduce stress-induced strain in the stiffer collagen-rich regions by undergoing greater deformation. Aside from a role in mechanical properties of the matrix, elastin may also affect cardiomyocyte physiology for its signaling role in inhibiting cell proliferation [31].

### Basement membrane is less extensive in the SAN

In contrast with the fibrous proteins, the basement membrane-associated and network-forming collagens IV, VI, and XVIII (or multiplexin) and glycoproteins were much less abundant in the SAN than the contractile LV. In fact, very few non-fibrillar type collagens and glycoproteins were detected in the SAN (Figs 2 and 3), indicating the basement membrane of the endomysium may be less extensive than that of LV. The SEM images corroborate with the mass spectrometry data such that a mesh-like basement membrane surrounding the contractile cardiomyocytes in the LV was not observed in the SAN. This is not to suggest that the basement membrane is absent in the SAN, because basement membrane-associated adhesion proteins, such as collagen IV, laminin and fibronectin, were present in the endomysial layer surrounding the pacemaking cardiomyocytes as shown by immunostaining (Figs 4–6). The basement membrane may be less extensive and lost during the decellularization process. Basement membrane is especially important in cell fate regulation and phenotype maintenance through integrin signaling as well as embedded growth factors [32]. In particular, fewer adhesive ECM proteins present in the SAN means fewer ligands for integrin binding and formation of focal adhesion sites, thus, less direct force transmission to the pacemaking cardiomyocytes. Mechanical force transduction has been shown to correlate with cardiomyocyte hypertrophy and myofilament development [33]. The implication of less force transmission to the cardiomyocytes in the SAN is in agreement with generally observed smaller cell size along with fewer and less developed myofilaments in the pacemaking cardiomyocytes [6].

### The SAN matrix scaffold may provide a protective niche for the pacemaking cardiomyocytes

**Fig 7:**
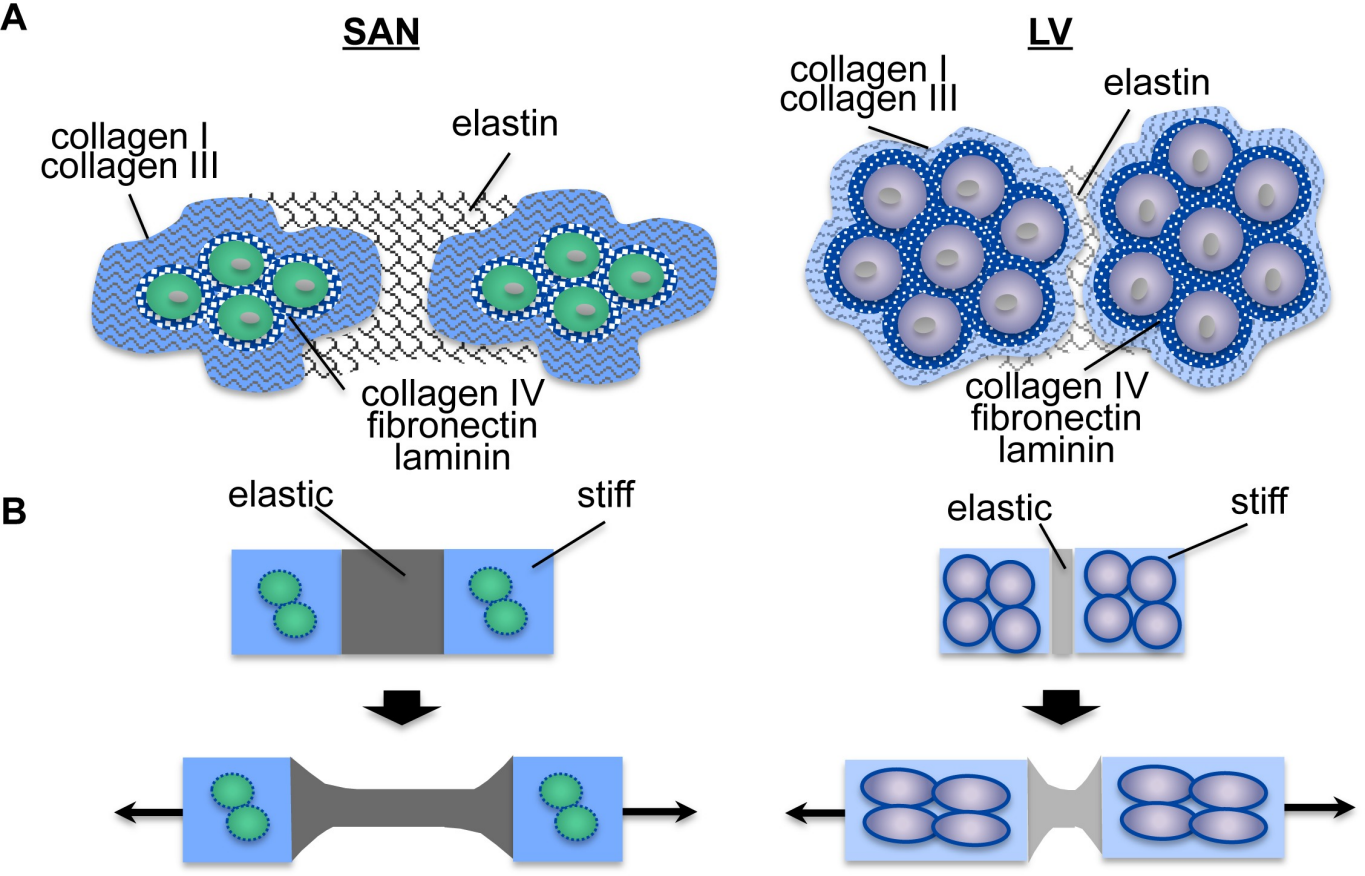
Model for SAN ECM protection of pacemaking cardiomyocytes. **A)** A schematic diagram reflecting the ECM protein distribution of the SAN with a more expansive perimysium composed of collagen I, collagen III, and elastin and a less developed basement membrane in the endomysium than that of the LV. A darker shade of colors reflects the abundance of ECM proteins detected in the SAN by mass spectrometry relative to the LV. **B)** A schematic diagram of (A) as a composite material with ‘elastic’ and ‘stiff’ regions responding to tensile stress. Our model suggests that the ‘elastic’ regions with an abundance of elastin would undergo deformation more readily rather than the ‘stiff’ regions of fibrillar collagens. Pacemaking cardiomyocytes in the SAN with a less developed basement membrane enclosed in a more expansive ‘stiff’ shell that is connected to more expansive neighboring ‘elastic’ regions would experience less mechanical strain with reduced mechanotransduction than that of the LV with a less expansive ‘stiff’ and ‘elastic’ regions, suggesting a possible protective action of ECM scaffold in the SAN against mechanical stress.

The pacemaking cardiomyocytes have long been hypothesized to be protected from mechanical stress induced by continuous cardiac contractions due to its strategic location at the epicardial junction between the intercaval region and the crista terminalis [6]. Our SAN ECM data is consistent across ultrastructural, mechanical, proteomic, and histological analyses. Collectively, they suggest that pacemaking cardiomyocytes are surrounded by stiffer and more abundant fibrillar collagens. This fibrillar collagen composition yields an overall stiffer and denser matrix scaffold as indicated by the higher Young’s modulus of decellularized SAN than the LV. However, areas abundant in elastic fibers are also present surrounding the cell clusters creating a heterogeneous ECM with regions of differing mechanical properties. Based on our SAN ECM data, we propose a composite material model to which the SAN ECM may serve as a protective scaffold for resident pacemaking cells that reduces the mechanical stress from continuous contractions (Fig 7). The denser and fibrillar matrix formed by load-bearing collagen I and III creates a protective shell with great tensile strength around the pacemaking cardiomyocytes, while the elastic fibers composed of elastin dissipate mechanical stress by undergoing deformation in reducing the mechanical strain of the pacemaking cardiomyocytes.

Aside from the biochemical composition and distribution that contribute to the protection of a stiffer matrix for the SAN resident cells, the orientation of the fibrous proteins also contributes to the stiffness as well. While cardiomyocytes were uniformly aligned in layers of the LV, as demonstrated by the cross-sectional and longitudinal endomysial immunostaining of ECM proteins, the organization in the SAN is less apparent (Figs 4–6, S3 Fig). Notably, the SAN ECM organization actually had greater similarities with the connective tissue on its epicardial side than the contractile LV or its neighboring contractile atrial cardiomyocytes in the crista terminalis of the right atrium (S4 Fig). This is in agreement with observations of a more uniform fiber alignment within the contractile tissue but a high disarray of matrix fiber orientation within the SAN, as reported by Ambrosi *et al*. using optical coherence tomography [34]. Interestingly, the epiphyseal plate in rabbits shows a matrix that is high in Young’s modulus with abundance of fibrillar collagens arranged in random orientation to protect the germinal cells compared to the hypertrophic zone [35]. A higher randomness in matrix fiber orientation may also be contributing to the stress bearing property in the SAN.

In addition to structural protection, the force transmission to pacemaking cardiomyocytes can be further minimized by a less developed basement membrane with fewer ligands available for integrin binding. Indeed, cardiomyocyte hypertrophy and myofilament development have been shown to directly correlate with the level of imposed mechanical stress [33]. While the SAN ECM can withstand greater tensile stress, fewer proteoglycans that are decorated with glycosaminoglycans (GAGs) known to resist compressive forces were detected compared to its LV counterpart. Although a compressive cushion may be reduced in the SAN, it may be sufficient to counter a systolic pressure, which is over 30 times lower in the right atrium where the SAN is located than that of LV. The phenotype of pacemaking cardiomyocytes—smaller cell size and underdeveloped myofilaments—may be due to their protection from mechanical strain by the surrounding ECM.

The unique properties of the SAN ECM may be partly attributed to resident fibroblasts. Fibroblasts are the main producers of ECM proteins and their number and phenotype are critical determinants of the resulting ECM properties. Fibroblasts are known to be present in the SAN in greater number [6, 7, 36] and likely contribute to the greater abundance of ECM proteins observed. In addition, fibroblasts respond to the mechanical environment such that stretch can induce increased synthesis of ECM proteins. Overstimulation from most pathological conditions, such as myocardial infarction, aortic stenosis, diabetic cardiomyopathy, and hypertrophic cardiomyopathy, can transdifferentiate these ECM-producing cells into myofibroblasts that augment the level of ECM production, resulting in fibrosis and stiffening of the heart [37, 38]. Furthermore, ECM homeostasis is balanced by matrix metalloproteinases (MMPs) that can breakdown the ECM and tissue inhibitors of metalloproteinases (TIMPs), both of which are produced by fibroblasts [39]. Notably, MMP2 has been reported to be high in the SAN head region compared to the atrial tissue and decreases with age and obesity [9]. This is consistent with reported downregulation of collagens I and III by 79% and elastin by 52% in the SAN of old and obese rats.

The human LV ECM has been shown to promote cardiomyocyte differentiation from human cardiac progenitors and mouse pluripotent stem cells [40, 41]. Hence, the SAN ECM described here may be utilized to preferentially direct progenitor fate and phenotype to that of pacemaking cardiomyocytes. Limited availability of human hearts for generation of human cardiac matrix scaffold for bioengineered pacemakers is one major bottleneck for tissue engineering; however, given the similarity of human and porcine extracellular scaffolds [20], the porcine cardiac ECM scaffolds investigated here may be a suitable clinical alternative. Moreover, the ECM properties of the SAN determined in this study provide a better understanding of the niche that is contributing to the pacemaking cardiomyocyte phenotype. The distinct properties of the SAN suggest that the LV matrix scaffolds currently used for tissue engineering of cardiac contractile tissue constructs may not be suitable for engineering biopacemakers due to insufficient tension resistance to protect the pacemaking cells. Considering that a biopacemaker is likely transplanted into a patient with compromised cardiac function and altered ECM as mentioned above, a suitable matrix scaffold bearing properties of healthy SAN ECM may be even more critical in sustaining the pacemaking function. The biomechanical and biochemical properties determined in this study present a set of criteria for which the synthetic matrices for bioengineered pacemakers may need to achieve to provide the appropriate physiological microenvironment.

## Supporting information

S1 Fig SAN isolation and decellularization. **A)** The SAN was identified in the right atrium at the junction between the crista terminalis and the intercaval region bordered by the superior and inferior vena cava (*left*). Hematoxyin and eosin (H&E) staining (*right*, *top*) showed the SAN region with minimal space occupied by the cardiac cells relative to the neighboring regions of atrial muscles. The SAN was manually dissected under dissecting scope prior to decellularization for SEM, AFM, and mass spectrometry analysis (*right*, *middle*). Both un-isolated and isolated SAN tissues appeared whitish and more opaque than the muscle area after decellularization (*right*, *bottom*). **B)** Nuclear stain with Hoechst 33342 was absent after decellularization, thus, verifying the completion of decellularization.
(TIF)

S2 Fig Cardiac tissue orientation. The SAN and LV were sectioned as specified in the diagram to yield cross-section and longitudinal sections used for imaging.
(TIF)

S3 Fig ECM protein staining in longitudinally sectioned SAN and LV tissues. ECM proteins (green) were co-stained with HCN4 (red) for pacemaking cardiomyocytes to identify the SAN. Although SAN showed some direction of alignment, the level of organization was less ordered than that of LV. Scale bar: 50 μm.
(TIF)

S4 Fig ECM protein staining of right atrium. The ECM protein staining (green) of the right atrial region that included the SAN marked by HCN4 staining (red) demonstrated the SAN matrix organization was actually more similar to the connective tissue (C) on the epicardial side (epi) than the atrial muscle region (M).
(TIF)

S5 Fig ECM protein staining of decellularized SAN and LV. Decellularized SAN and LV stained for ECM proteins (green) were comparable to those staining of native SAN and LV tissue in Figs 4–6.
(TIF)

S6 Fig Representative elastin peptide spectrum from GuHCl-insoluble fraction of the SAN. A representative tandem mass spectrum of a non-tryptic elastin peptide (GAGAIPGIGGIAGAGAPAAA from S1 Table) identified by LC-MS/MS is given with y (blue) and b (red) ions labeled.
(TIF)

S1 Table Elastin peptides identified by LC-MS/MS in elastin-digested GuHCl-insoluble fraction of SAN. A total of 11 unique elastase-derived peptides exclusively matching porcine elastin (Uniprot accession number: A0A097ZMY1-PIG) were identified with 19 total tandem mass spectra. No elastin peptides were identified in LV. Peptide sequence and associated identification metrics are provided (including theoretical and observed mass, charge, mass error, X! Tandem identification scores (-LogE (expect scores)), Scaffold peptide identification probabilities, and spectral counts. Hydroxylation of proline or n-terminal ammonia loss are indicated with bold, underlined letters and contribute a mass change of +16 or -17 Da respectively.
Primary sequence coverage of elastin (Uniprot accn# A0A097ZMY1) protein present in SAN was 26%. A protein BLAST of this protein reveals high sequence identity with other known elastin proteins, including: a 95.6% match with another pig elastin isoform (A0A097ZMY9), 76.1–79.4% match with bovine elastin isoforms 1–7 (P04985-2/3/4/5/6/7), a 69.2% match with human elastin (P15502), and a 67.5% match with mouse elastin (P54320).
(TIF)

S1 File Detailed methods for mass spectrometry and supplemental figures. (PDF)
