# Supplementary figures and images for: Biochemical and biomechanical properties of the pacemaking sinoatrial node extracellular matrix are distinct from contractile left ventricular matrix
Source: PLoS One. 2017 Sep 21;12(9):e0185125. doi: 10.1371/journal.pone.0185125 (PMC5608342; doi:10.1371/journal.pone.0185125)

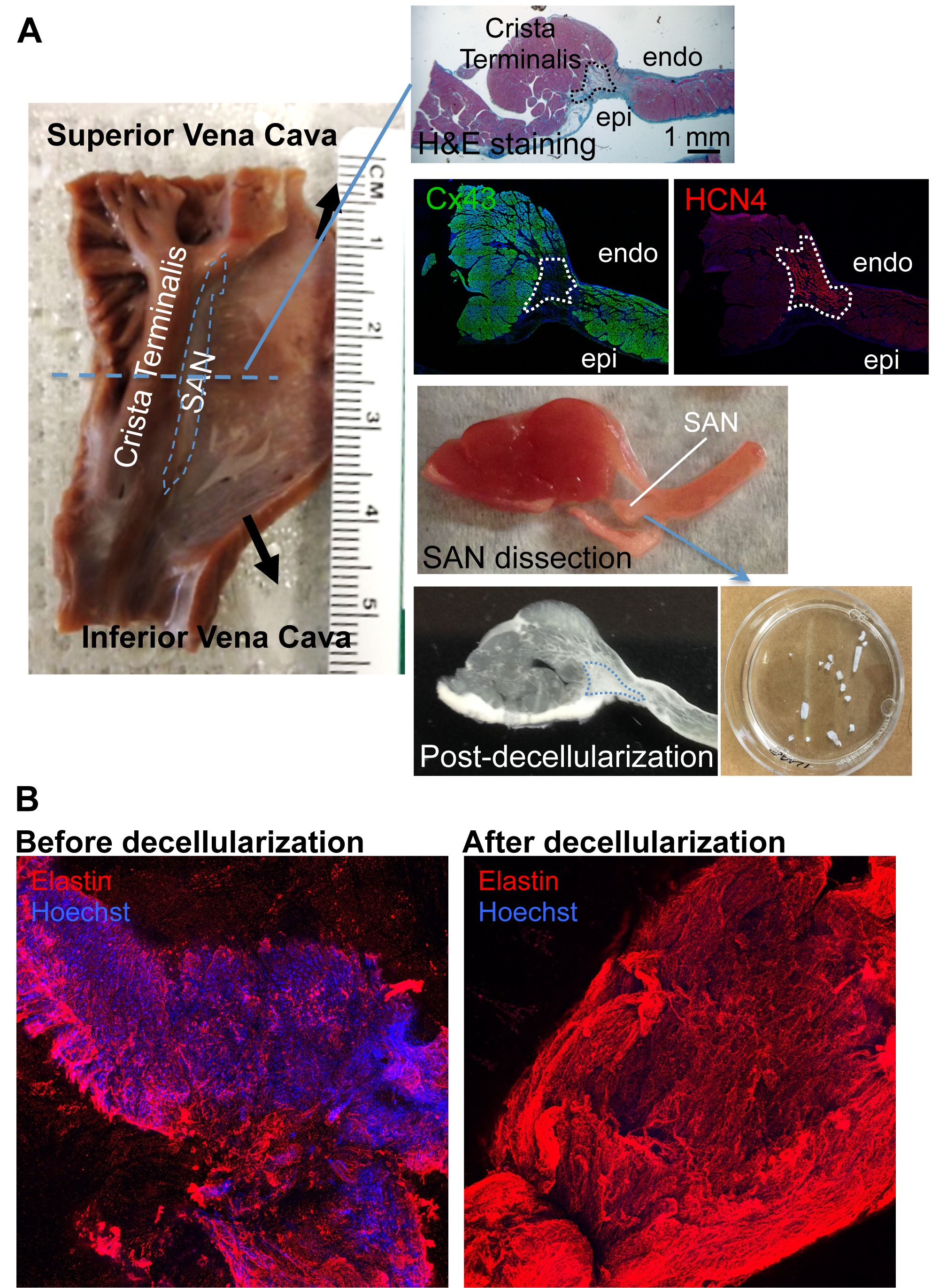

Supplement: S1 Fig — A) The SAN was identified in the right atrium at the junction between the crista terminalis and the intercaval region bordered by the superior and inferior vena cava (left). Hematoxyin and eosin (H&E) staining (right, top) showed the SAN region with minimal space occupied by the cardiac cells relative to the neighboring regions of atrial muscles. The SAN was manually dissected under dissecting scope prior to decellularization for SEM, AFM, and mass spectrometry analysis (right, middle). Both un-isolated and isolated SAN tissues appeared whitish and more opaque than the muscle area after decellularization (right, bottom). B) Nuclear stain with Hoechst 33342 was absent after decellularization, thus, verifying the completion of decellularization. (TIF) [file pone.0185125.s001.tif]

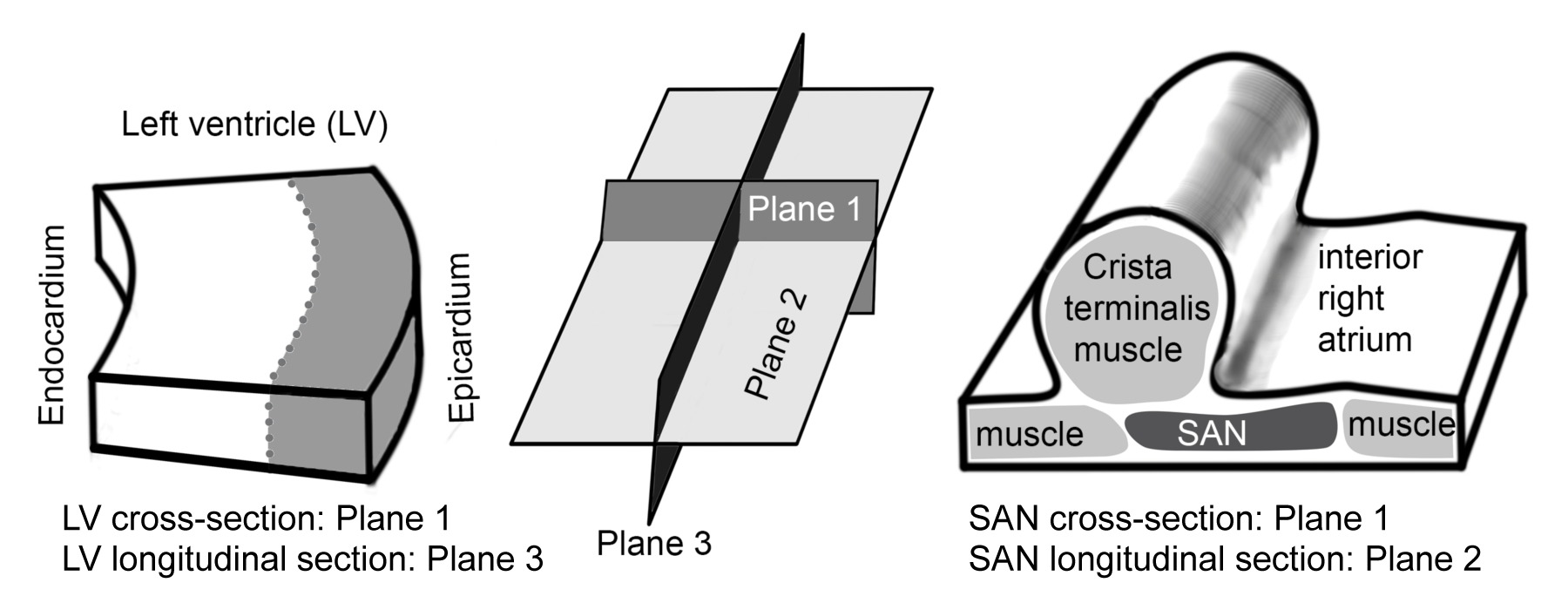

Supplement: S2 Fig — The SAN and LV were sectioned as specified in the diagram to yield cross-section and longitudinal sections used for imaging. (TIF) [file pone.0185125.s002.tif]

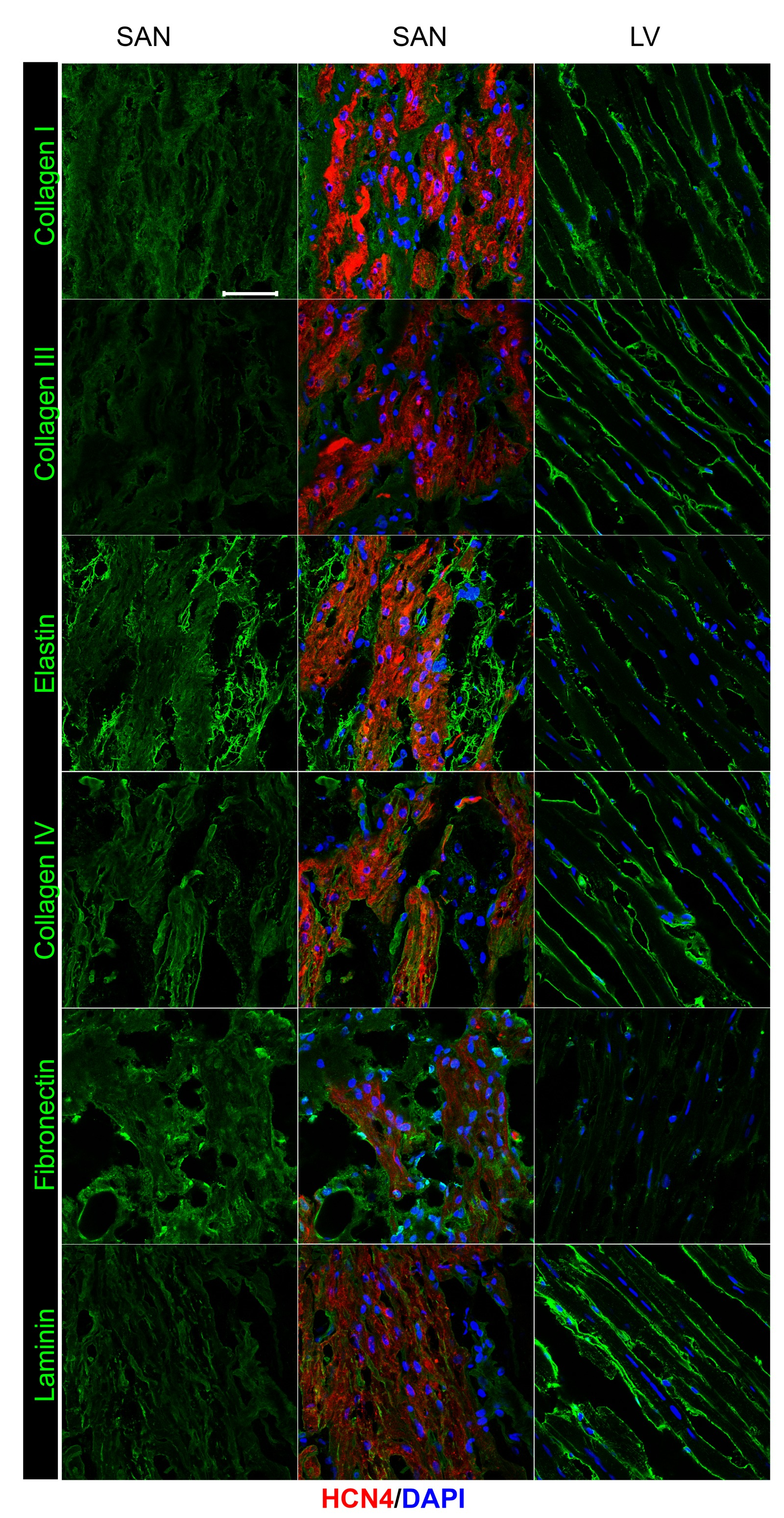

Supplement: S3 Fig — ECM proteins (green) were co-stained with HCN4 (red) for pacemaking cardiomyocytes to identify the SAN. Although SAN showed some direction of alignment, the level of organization was less ordered than that of LV. Scale bar: 50 μm. (TIF) [file pone.0185125.s003.tif]

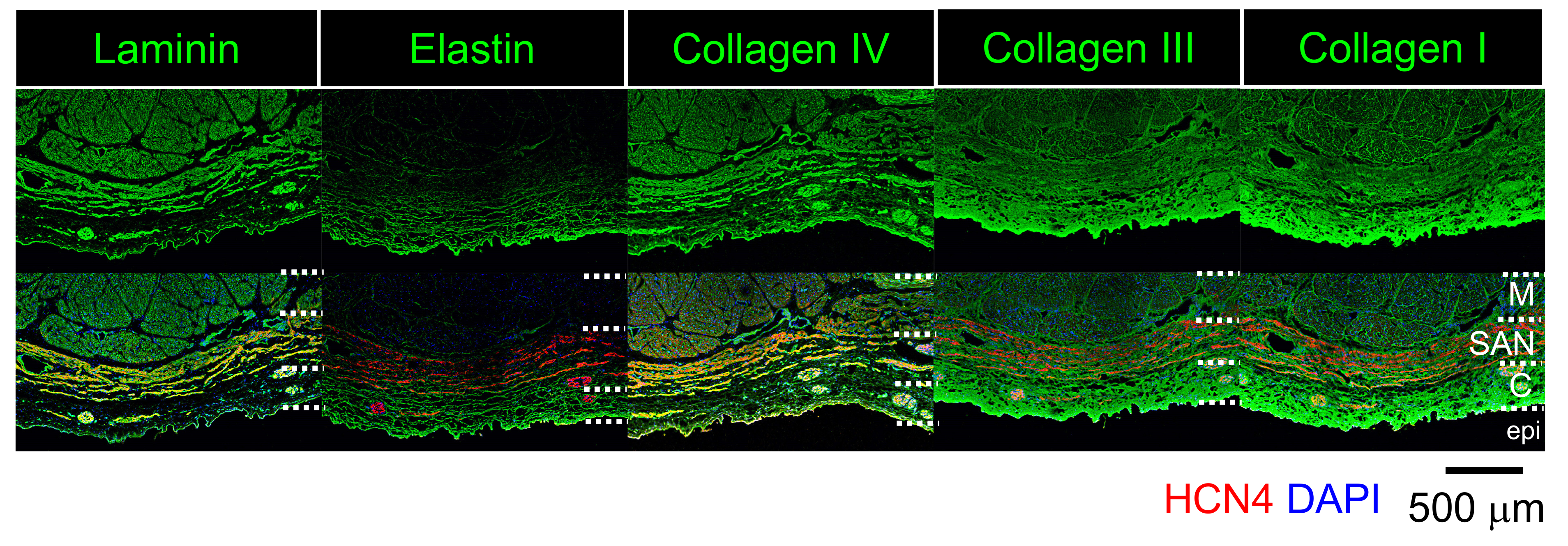

Supplement: S4 Fig — The ECM protein staining (green) of the right atrial region that included the SAN marked by HCN4 staining (red) demonstrated the SAN matrix organization was actually more similar to the connective tissue (C) on the epicardial side (epi) than the atrial muscle region (M). (TIF) [file pone.0185125.s004.tif]

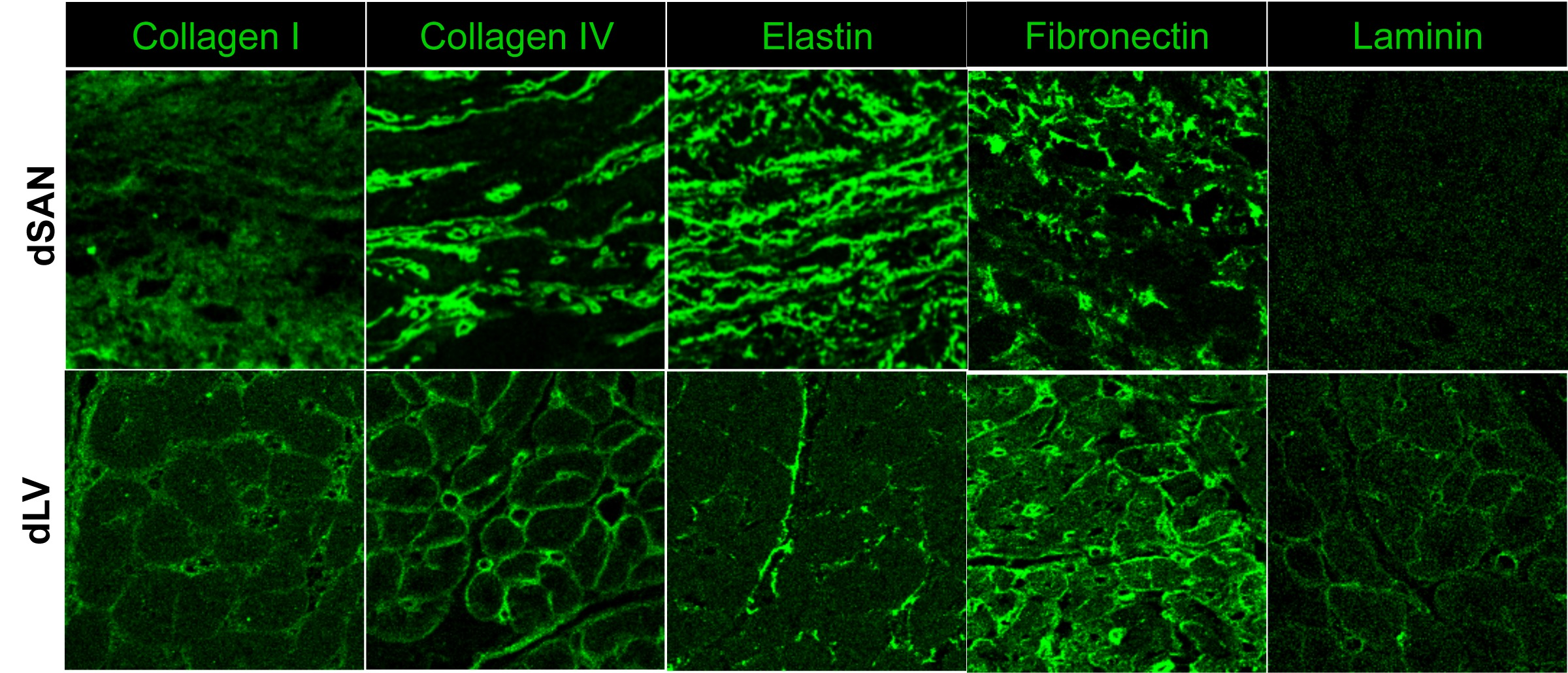

Supplement: S5 Fig — Decellularized SAN and LV stained for ECM proteins (green) were comparable to those staining of native SAN and LV tissue in Figs 4–6. (TIF) [file pone.0185125.s005.tif]

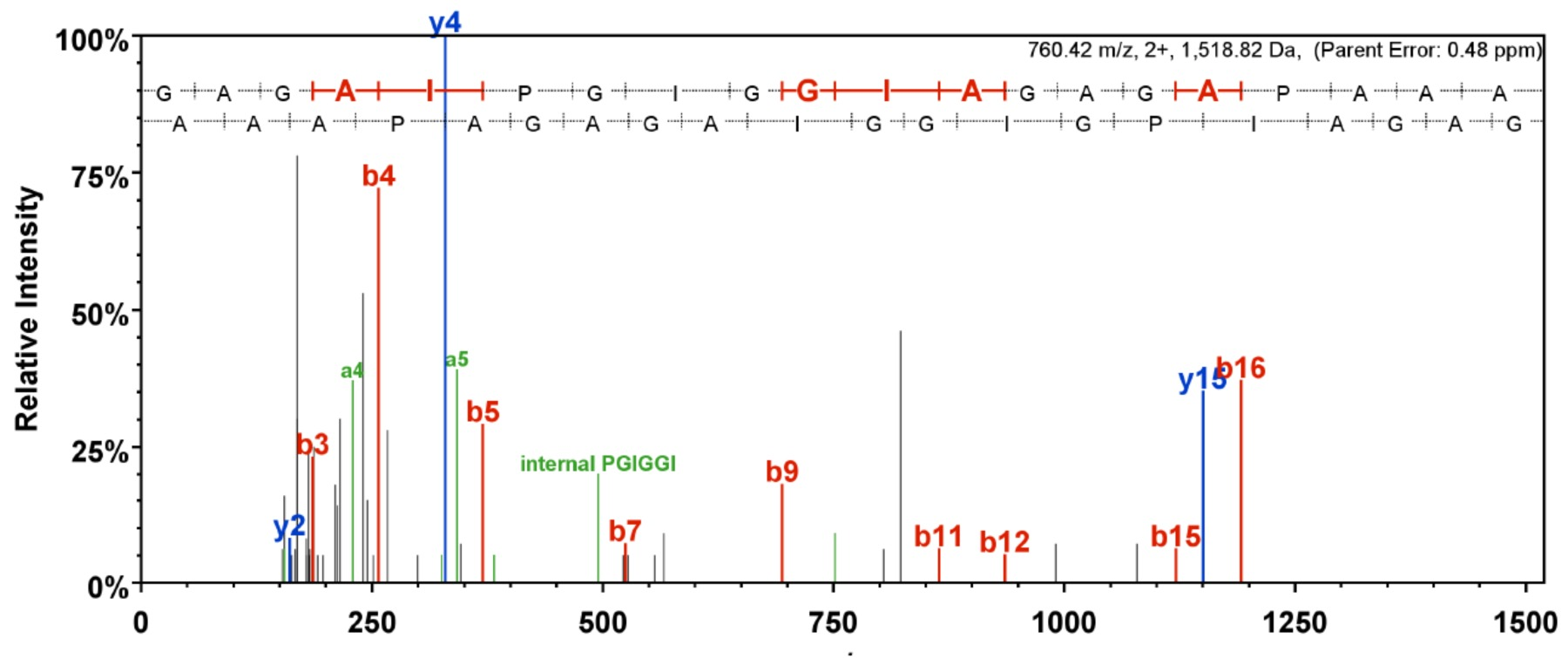

Supplement: S6 Fig — A representative tandem mass spectrum of a non-tryptic elastin peptide (GAGAIPGIGGIAGAGAPAAA from S1 Table) identified by LC-MS/MS is given with y (blue) and b (red) ions labeled. (TIF) [file pone.0185125.s006.tif]

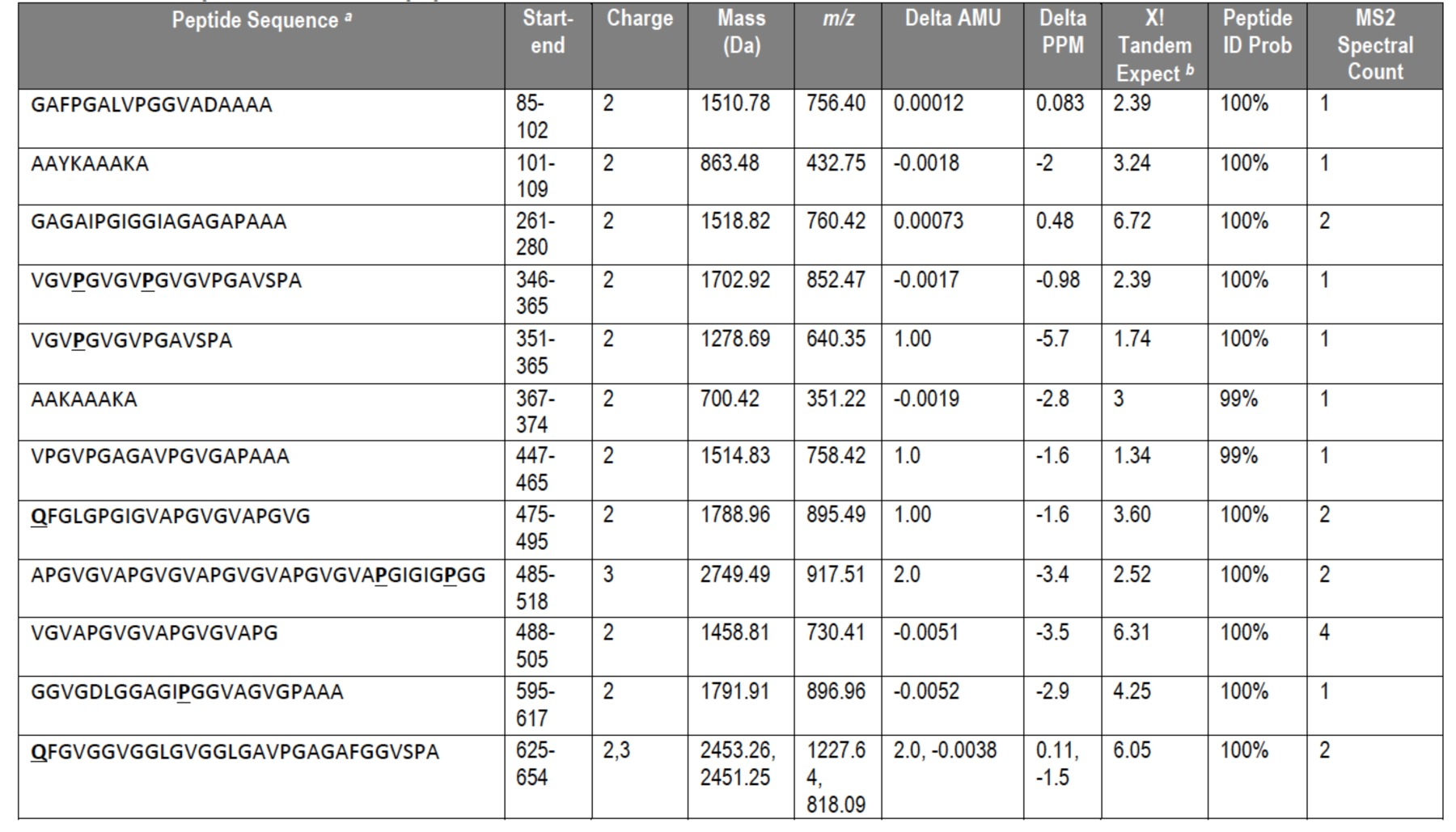

Supplement: S1 Table — A total of 11 unique elastase-derived peptides exclusively matching porcine elastin (Uniprot accession number: A0A097ZMY1-PIG) were identified with 19 total tandem mass spectra. No elastin peptides were identified in LV. Peptide sequence and associated identification metrics are provided (including theoretical and observed mass, charge, mass error, X! Tandem identification scores (-LogE (expect scores)), Scaffold peptide identification probabilities, and spectral counts. Hydroxylation of proline or n-terminal ammonia loss are indicated with bold, underlined letters and contribute a mass change of +16 or -17 Da respectively. Primary sequence coverage of elastin (Uniprot accn# A0A097ZMY1) protein present in SAN was 26%. A protein BLAST of this protein reveals high sequence identity with other known elastin proteins, including: a 95.6% match with another pig elastin isoform (A0A097ZMY9), 76.1–79.4% match with bovine elastin isoforms 1–7 (P04985-2/3/4/5/6/7), a 69.2% match with human elastin (P15502), and a 67.5% match with mouse elastin (P54320). (TIF) [file pone.0185125.s007.tif]
