# Supplementary material for: Biochemical and biomechanical properties of the pacemaking sinoatrial node extracellular matrix are distinct from contractile left ventricular matrix
Source: PLoS One. 2017 Sep 21;12(9):e0185125. doi: 10.1371/journal.pone.0185125 (PMC5608342; doi:10.1371/journal.pone.0185125)
Supplement: S1 File — (PDF) [file pone.0185125.s008.pdf]

SUPPLEMENTAL MATERIAL

Biochemical and biomechanical properties of the pacemaking sinoatrial node extracellular matrix are distinct from contractile left ventricular matrix

Jessica M. Gluck*[1], Anthony W. Herren*[2], Sergey Yechikov[1], Hillary Kao[1,3], Ambereen Khan[1,3], Brett S. Phinney[2], Nipavan Chiamvimonvat[1,4], James W. Chan[5,6], Deborah K. Lieu[1]**

1. Division of Cardiovascular Medicine, Department of Internal Medicine, University of California, Davis; Davis, CA, USA
2. UC Davis Genome Center, University of California, Davis; Davis, CA, USA
3. Bridges to Stem Cell Research Program, California State University, Sacramento; Sacramento, CA, USA
4. Department of Veterans Affairs, Northern California Health Care System, Mather, CA, USA
5. Center for Biophotonics, University of California, Davis; Sacramento, CA, USA
6. Department of Pathology and Laboratory Medicine, University of California, Davis; Sacramento, CA, USA

* denotes equal contribution

Short Title: Extracellular Matrix of the Sinoatrial Node

**To whom correspondence should be addressed:

Deborah K. Lieu, Ph.D.
Assistant Adjunct Professor
University of California, Davis
Department of Internal Medicine
Division of Cardiovascular Medicine
Institute for Regenerative Cures 1616
2921 Stockton Blvd.
Sacramento, CA 95817
Tel: 916-734-0683
Email: dklieu@ucdavis.edu

SUPPLEMENTAL MATERIAL

S1 File. Detailed methods for mass spectrometry

Based on a modified method,[13] 150mg wet weight of cardiac ECM was prepared from pig hearts decellularized with 1% SDS extraction and DNAse1 treatment. Tissue was manually minced and soluble protein extracted for 72 hrs at RT with vigorous shaking in guanidine-hydrochloride (GuHCl) extraction buffer (in mol/L: 4 GuHCl, 0.05 sodium acetate, 0.025 EDTA, pH 6.8). Samples were centrifuged at 16,000 x g for 30 min to separate the GuHCl-soluble (GuHCl-S) and GuHCl-insoluble (GuHCl-IS) protein fractions.

Soluble protein from the GuHCl-S fractions was precipitated with 9X volume of ethanol for 1 hr at -20°C and centrifuged at 16,000 x g for 30min at 4C. The resulting pellets were air-dried, resuspended in de-glycosylation buffer (in mmol/L: 100 NaCl, 60 sodium acetate, pH 6.8), and reacted with 0.05 units each of chondroitinase ABC and keratanase (Sigma) to remove glycosaminoglycans prior to mass spectrometry analysis. Protein concentrations were measured by the bicinchoninic acid assay (Thermo Pierce) and 50 ug of soluble protein was precipitated from solution (ProteoExtract, Calbiochem) and air dried. GuHCl-S protein pellets were resuspended in 6 M urea/50 mM Tris-Cl pH 8.8, reduced in 5 mM DTT, and alkylated in 15 mM iodoacetamide. Protein was proteolytically digested in solution with sequencing grade Lys-C/trypsin (Promega) at an enzyme:protein w/w of 1:15 for 4 hrs at 37 °C, diluted to 0.8 M urea with 50mM Tris-Cl pH 7.5, and digested another 14 hrs overnight at 37°C. GuHCl-IS samples were resuspended as above, halved, and reacted with 12ug of either LysC-trypsin as above or elastase (Promega) in 2 M urea/50mM Tris-Cl pH 8.8. Undigested material was cleared by centrifugation and supernatants acidified with trifluoroacetic acid (TFA) to a final concentration of 0.5%.

Digested peptides were concentrated and desalted with C18 resin (Macro Spin Column, Nest Group), eluted with 60% acetonitrile/0.1% TFA, lyophilized in a vacuum concentrator and reconstituted in 2% acetonitrile/0.1% TFA for LC-MS/MS. Peptide concentrations were measured using the Pierce fluorometric peptide assay (Thermo Pierce) and 2 μg of total peptide was separated on an EASY-nLC 1200 UHPLC with a custom Proxeon nanospray source outfitted with a column oven heated to 35°C. Peptides were loaded on a 100 μm x 25 mm Magic C18 100Å 5U reverse phase trap before being separated using a 75 μm x 250 mm Magic C18 200Å 3U reverse phase column packed in house. Peptides were eluted with an increasing percentage of acetonitrile over the course of a 180-minute analytical gradient with a flow rate of 300 nl/min. Mass spectra were acquired on a Thermo Scientific Exactive Plus Orbitrap Mass Spectrometer (Thermo Fisher Scientific) in positive ion, data-dependent mode with one MS precursor scan followed by 15 MS/MS scans. MS spectra were obtained for an m/z range of 350-1600, acquired with a resolution of 70,000, and an AGC target of $1x10^6$ ions or a maximum injection time of 30 msec. MS/MS spectra were acquired using a top 15 method where the top 15 ions in the MS spectra were subjected to HCD (High Energy Collisional Dissociation). MS/MS spectra were acquired with a resolution of 17,500, an AGC target of $5x10^4$ ions or a maximum injection time of 50 msec. A mass isolation window of 1.6 m/z was used for precursor ion selection, charge states 2-4 were accepted, and a normalized collision energy of 27% was used for fragmentation. A 5 sec duration was used for dynamic exclusion.

Tandem mass spectra were extracted and charge state deconvoluted with Proteome Discoverer 1.4 (Thermo Scientific) and searched using the Andromeda (MaxQuant 1.5.3.3 framework, Max-Planck Institute) or X! Tandem (The GPM, thegpm.org; Vengeance 2015.12.15.2) peptide spectrum matching algorithms against a Sus scrofa protein database (uniprot.org; September 8 2015). Both search algorithms were configured to search all proteins in forward and reverse orientation plus the cRAP database of common laboratory contaminants (www.thegpm.org/crap; 114 entries) totaling 70,506 entries. For all

searches, carbamidomethylation of cysteine was specified as a fixed modification. Deamidation of asparagine and glutamine, oxidation of methionine and tryptophan, hydroxylation of lysine and proline, Glu->pyro-Glu, Gln->pyro-Glu, and ammonia loss of the n-terminus were specified as variable modifications. Identified proteins were annotated with gene ontology (GO) terms (uniprot.org, downloaded September 8 2015) and ECM protein contribution was determined by filtering identified proteins for ECM GO terms, as well as by manual curating.

MaxQuant was configured to search a strict trypsin digest with 3 max missed cleavages, a minimum peptide length of 7, and a maximum of 5 modifications per peptide. Orbitrap fourier transform mass spectrometry (FTMS) was used for MS/MS with parent and fragment mass errors of 20 PPM. A target decoy based approach was used to filter the data to achieve peptide and protein level false discovery rates of 1%. All proteins were identified with at least 1 unique peptide and peptides were assigned in a razor peptide fashion, where every non-unique razor peptide is assigned to the protein group with the most other peptides and is used only once. For label free quantification, individual protein abundance within each sample was calculated using Intensity Based Absolute Quantification (IBAQ) and taken as a percentage of the total summed ECM within each sample.

X! Tandem was used to search elastase digests and was configured for non-specific enzyme cleavage with parent and fragment ion mass tolerances of 20 PPM. Scaffold (version 4.4.0, Proteome Software Inc., Portland, OR) was used to validate MS/MS based protein and peptide identifications with a target/decoy based approach. Peptide and protein identifications were accepted if they could be established at greater than 95% probability to achieve an FDR less than 1% for peptide and 5% for protein by the Scaffold Local FDR algorithm. Protein identifications contained at least 2 identified peptides. Protein probabilities were assigned by the Protein Prophet algorithm within Scaffold. Proteins that contained similar peptides and could not be differentiated based on MS/MS analysis alone were grouped to satisfy the principles of parsimony. Proteins sharing significant peptide evidence were grouped into clusters. We report here only those proteins for GuHCl-IS elastase digests that were not identified in corresponding tryptic digests and IBAQ values were not calculated for these non-tryptic digests. Proteotypic elastin MS/MS data were manually validated and required exclusive, unique peptides with X! Tandem identification –LogE (expect scores) greater than 1.2 and mass accuracy of $\leq \pm 5$ppm. Elastin protein sequence conservation across species was determined by the BLASTP algorithm (uniprot.org and blast.ncbi.nlm.nih.gov) and in silico trypsin cleavage probabilities calculated with PeptideCutter (expasy.org).
